# Supplementary figures and images for: Region-specific H3K9me3 gain in aged somatic tissues in Caenorhabditis elegans
Source: PLoS Genet. 2021 Sep 10;17(9):e1009432. doi: 10.1371/journal.pgen.1009432 (PMC8457455; doi:10.1371/journal.pgen.1009432)

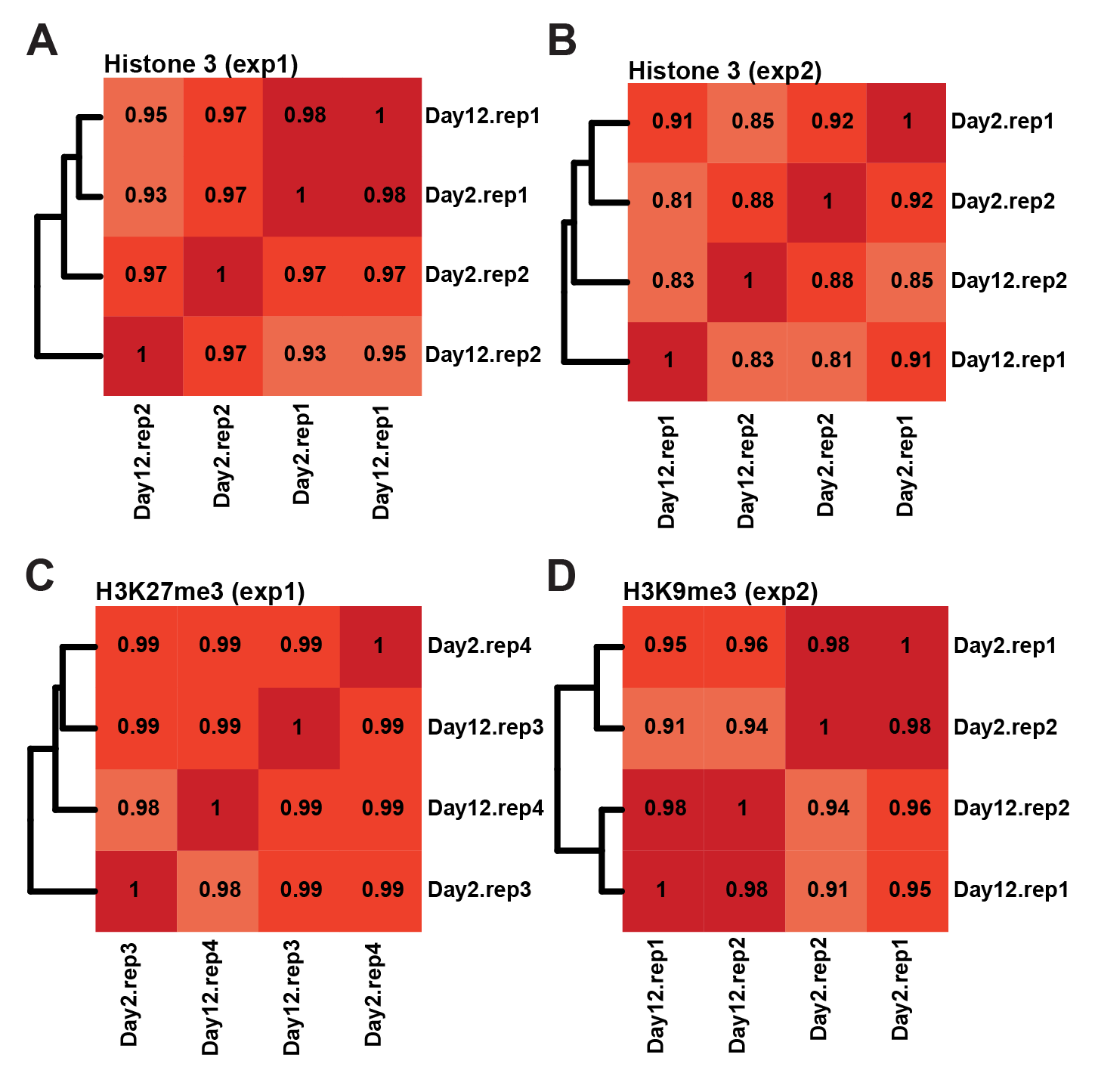

Supplement: S1 Fig — Biological replicates of individual ChIP-seq experiments were highly correlated. Pair-wise Pearson’s correlation coefficient was calculated using genome-wide tag coverage in 15kb sliding windows. There were two independent H3 control and treatment ChIP-seq experiments, H3K27me3 (A and C) and H3K9me3 (B and D). (TIF) [file pgen.1009432.s001.tif]

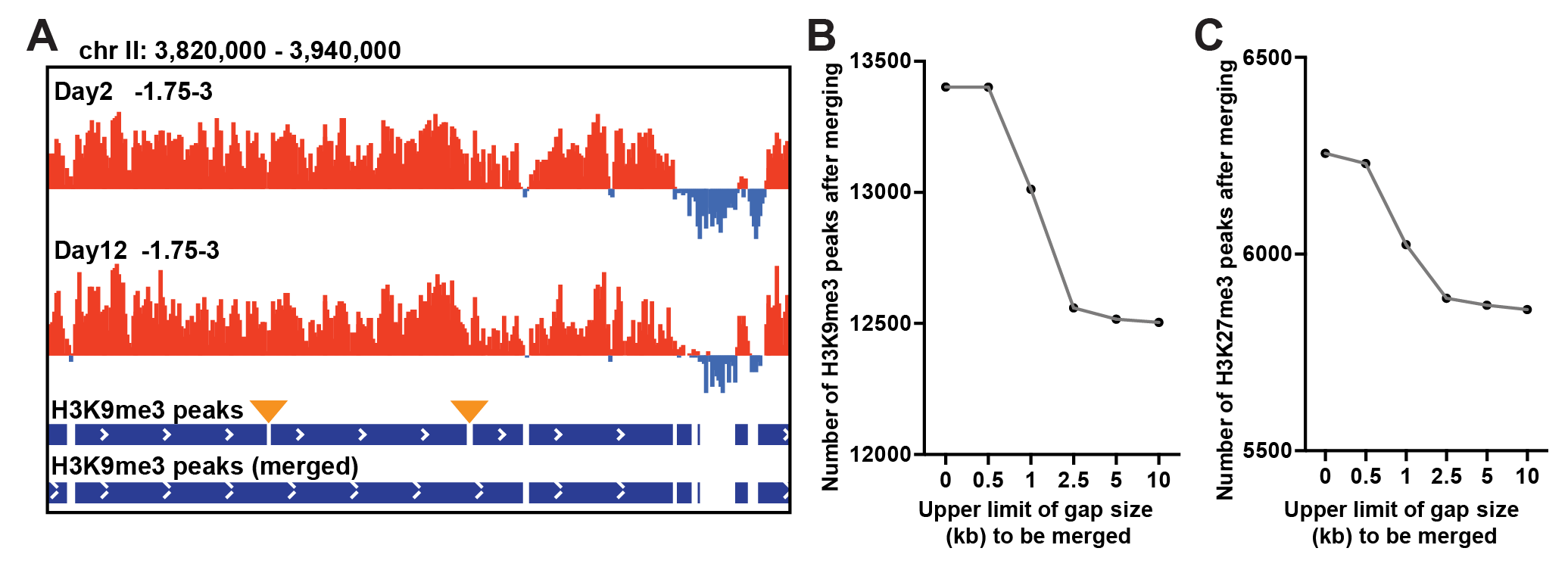

Supplement: S2 Fig — (A) IGV browser screenshot showing z-scores of H3K9me3 signal intensity normalized to H3 in day 2 and day 12 adults (top). Neighboring peaks separated by gaps with a positive mean z-score (yellow wedges) were merged together (bottom). The numbers on the top left corner of each track indicate the y-axis range. (B) The number of H3K9me3 (B) or H3K27me3 (C) peak regions after merging neighboring peaks with a varying maximum threshold of gap size. (TIF) [file pgen.1009432.s002.tif]

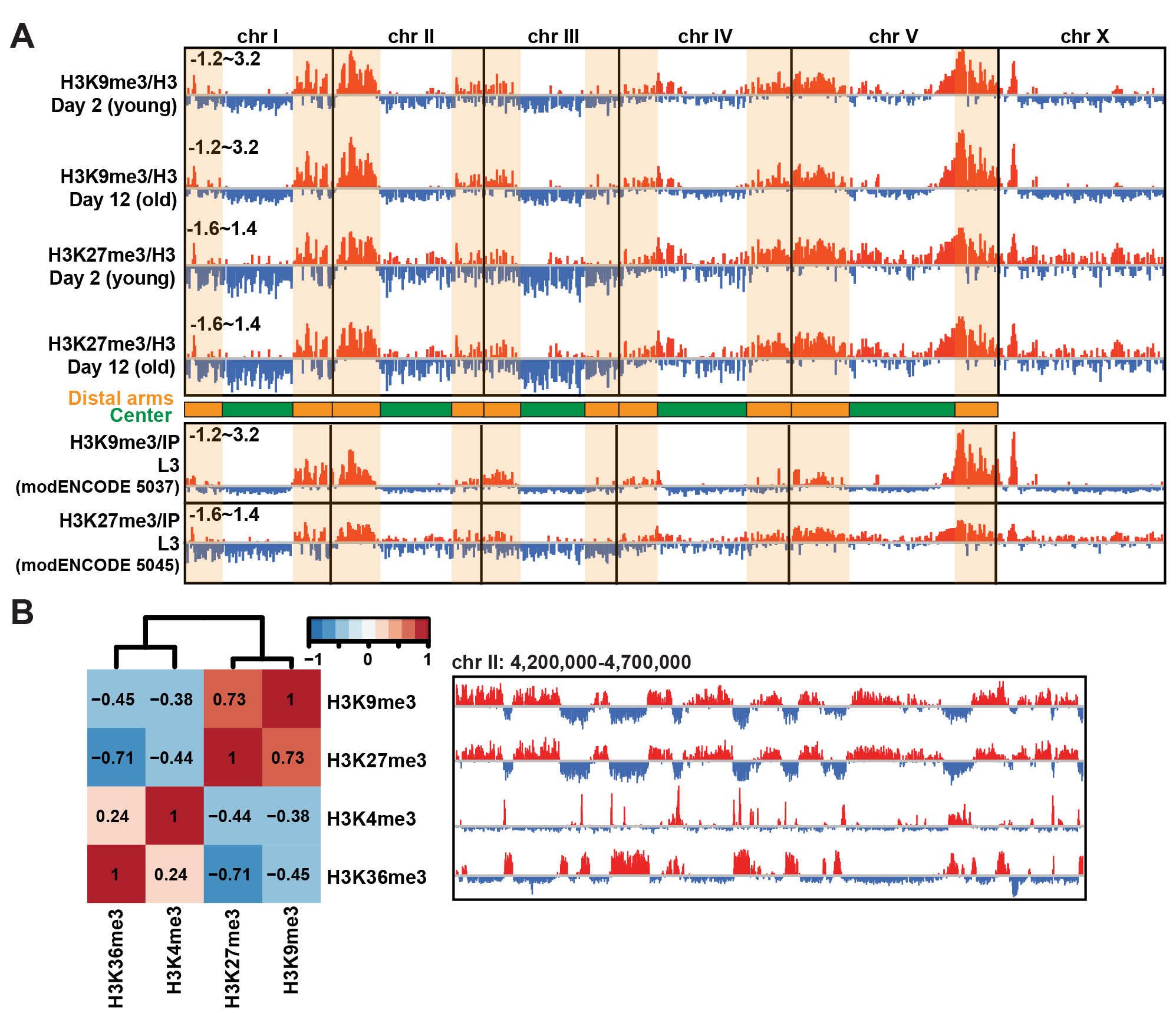

Supplement: S3 Fig — (A) H3K9me3 and H3K27me3 were enriched in the distal arm regions of chromosomes. IGV browser view showing the z-score of normalized H3K9me3 or H3K27me3 signal intensity in 20-bp bins across the entire genome. The top four tracks showed the ChIP-seq signal intensity normalized to H3 in young (day 2) and old (day 12) glp-1(e2141) adults from data sets in this study. The bottom two tracks showed the ChIP-seq signal intensity normalized to input DNA in N2 L3 larvae from data sets generated by the modENCODE (5037 and 5045). Red and blue bars indicate positive and negative, respectively, z-score. The numbers on the top left corner of each track indicate the y-axis range (z-score). Distal arms of autosomes are highlighted by the orange shaded regions. (B) The genome-wide distribution of active (H3K4me3 and H3K36me3) and repressive (H3K9me3 and H3K27me3) histone modifications were often mutually exclusive. Heatmap showing pair-wise Pearson’s correlation coefficient between the genome-wide distribution of peak regions of active and repressive histone modifications in adult worms (left). IGV browser screenshot showing the z-scores of H3-normalized signal intensity of active and repressive histone marks (right). (TIF) [file pgen.1009432.s003.tif]

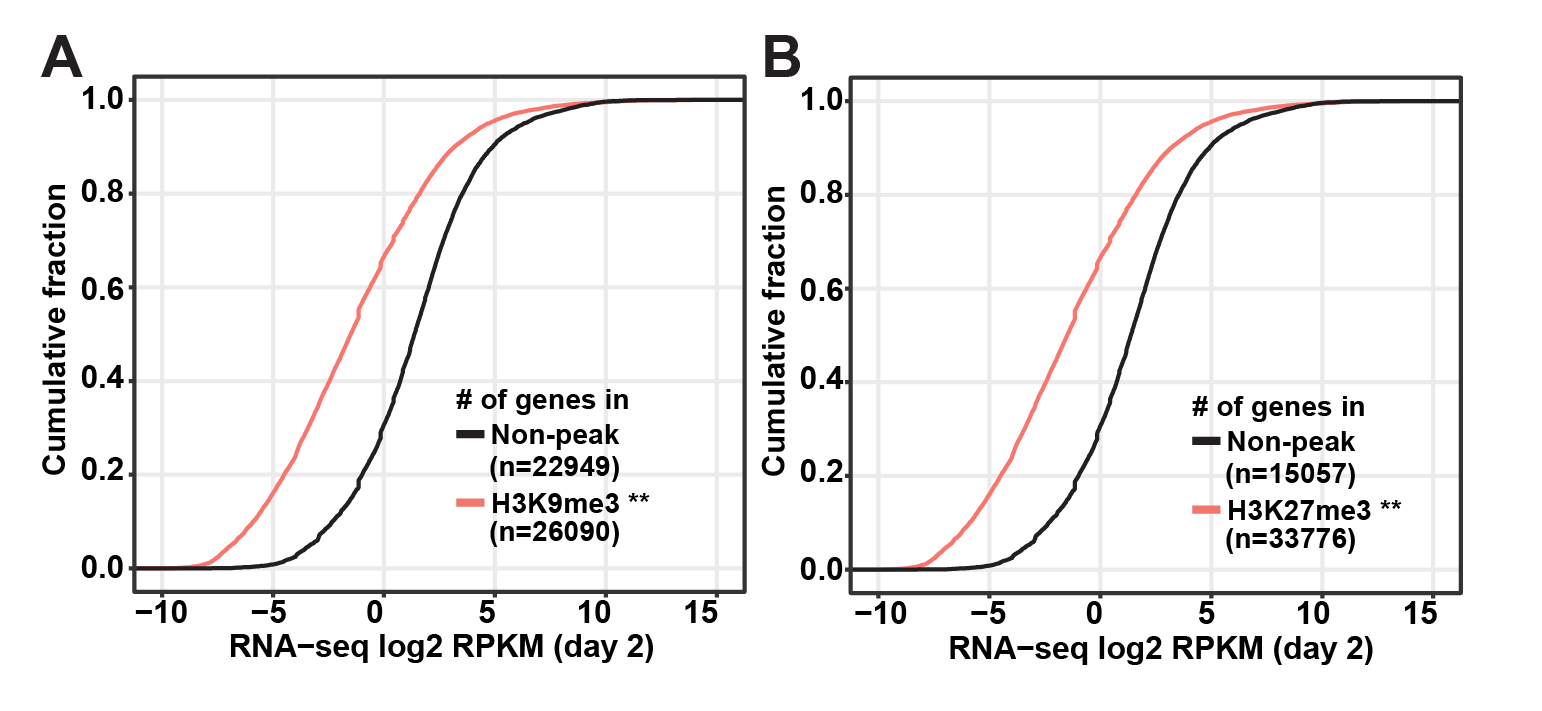

Supplement: S4 Fig — (A-B) Cumulative frequency plots of the normalized RNA read counts (log2 RPKM) of genes associated with repressive peak regions (red curves) or non-peak regions (black curves). There were net negative changes in the RNA expression levels of genes in H3K9me3 (A) or H3K27me3 (B) peak regions (KS test: **, p-value < 0.0001). (TIF) [file pgen.1009432.s004.tif]

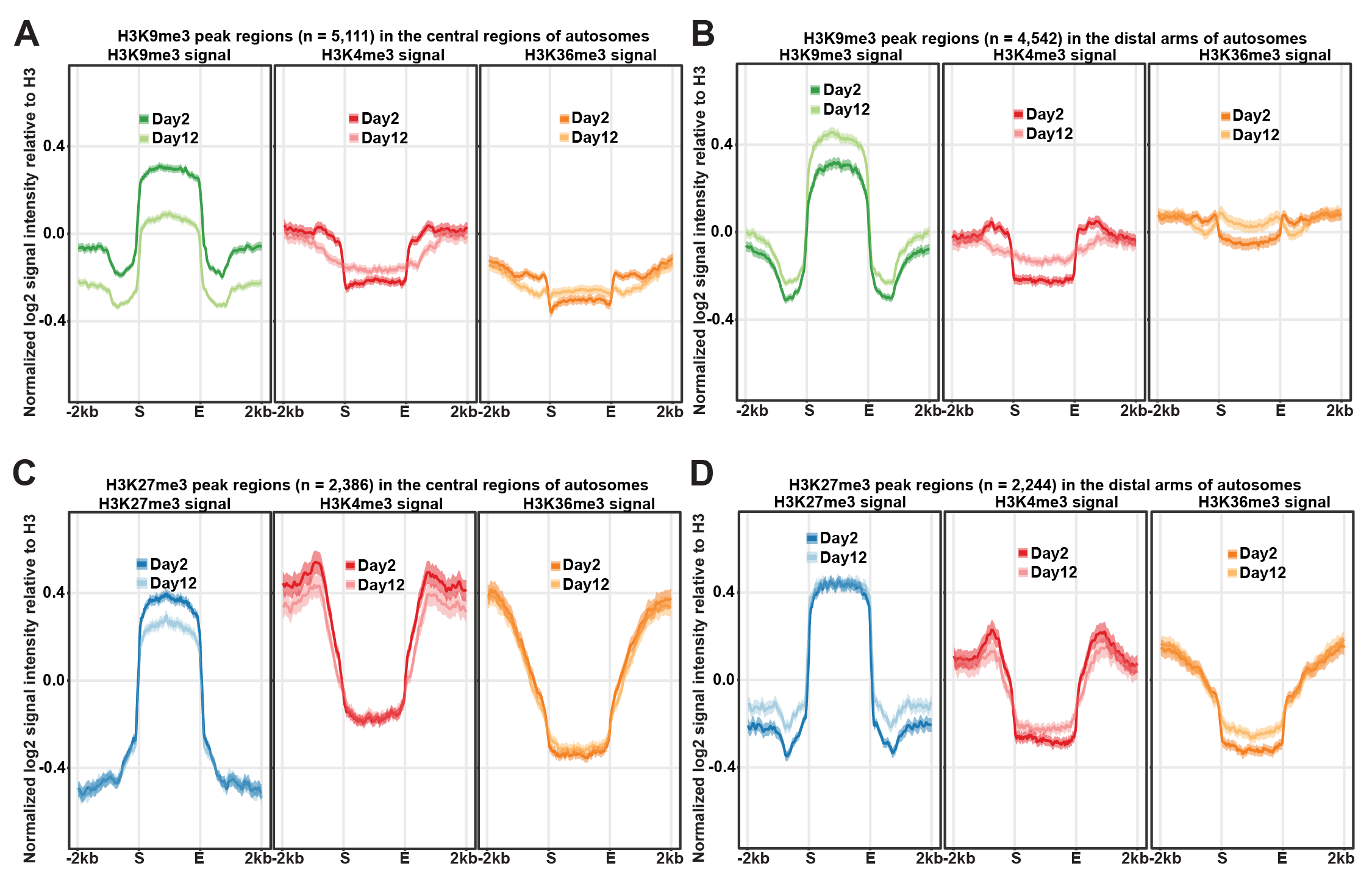

Supplement: S5 Fig — Metaplots showing the normalized ChIP-seq signal intensity (z-score) of individual histone marks (H3K9me3, H3K27me3, H3K4me3 or H3K36me3) in young (day 2) and old (day 12) germlineless glp-1(e2141) mutants. Peak regions in individual panels are H3K9me3 peaks in the central regions of autosomes (A), H3K9me3 peaks in the distal arms of autosomes (B), H3K27me3 peaks in the central regions of autosomes (C), and H3K27me3 peaks in the distal arms of autosomes (D). There was age-dependent loss of repressive marks in the central regions and gain of H3K9me3 in the distal arms of autosomes. (TIF) [file pgen.1009432.s005.tif]

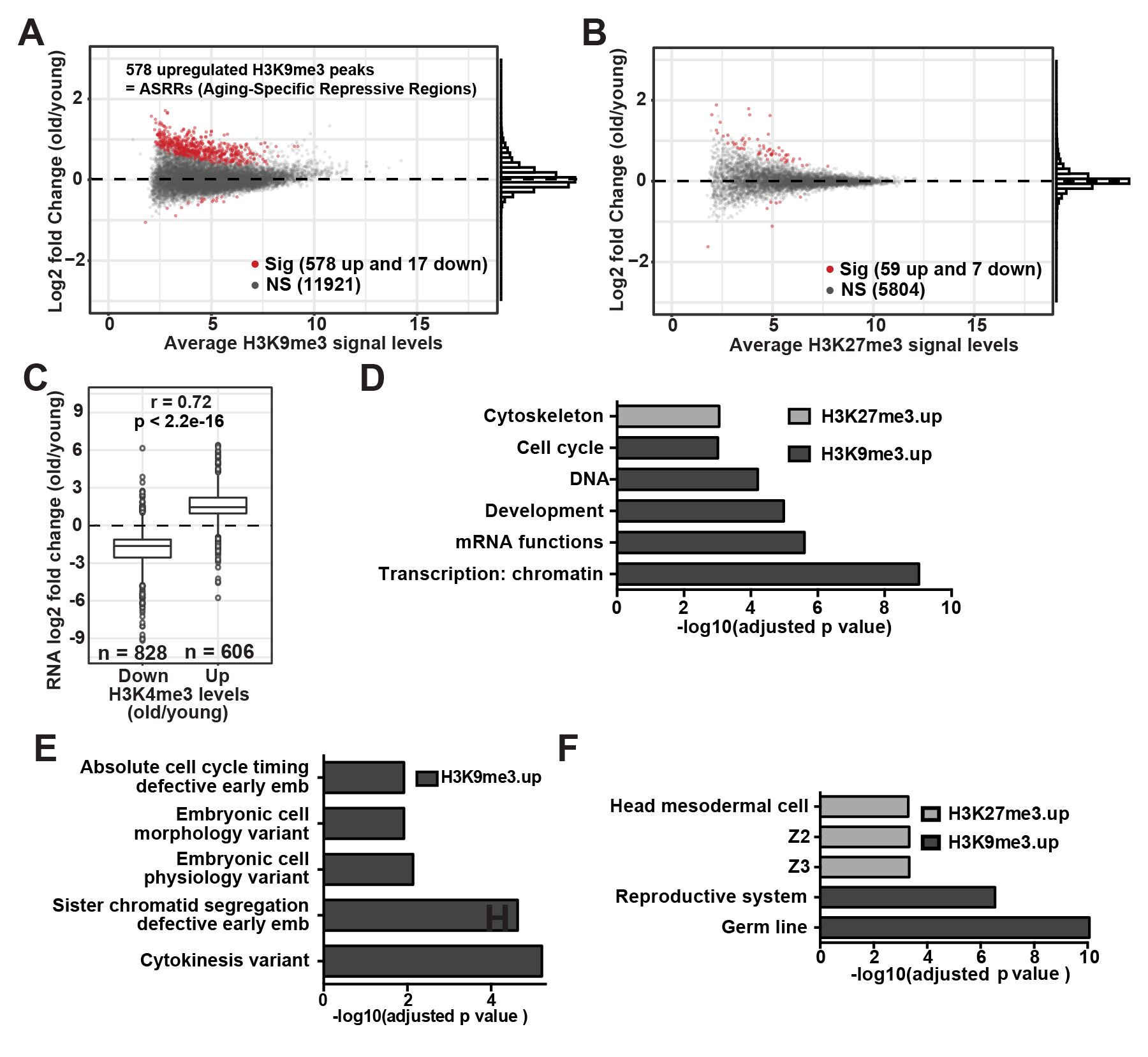

Supplement: S6 Fig — MA-plot showing log2 fold changes of H3K9me3 (A) and H3K27me3 (B) ChIP-seq read counts in peak regions with age (old/young) in germlineless glp-1(e2141) worms. Peaks with significant changes are highlighted in red (Sig), and the rest are colored in grey (NS, not significant). (C) Boxplots of the RNA log2 fold change of differentially expressed genes associated with differential H3K4me3 peaks. RNA expression changes strongly correlated with H3K4me3 changes (Pearson’s correlation coefficient = 0.72). (D-F) Enrichment analyses of functional terms (D), phenotypes (E), and tissue types (F) were performed on the genes associated with differential H3K9me3 and H3K27me3 peak regions. (TIF) [file pgen.1009432.s006.tif]

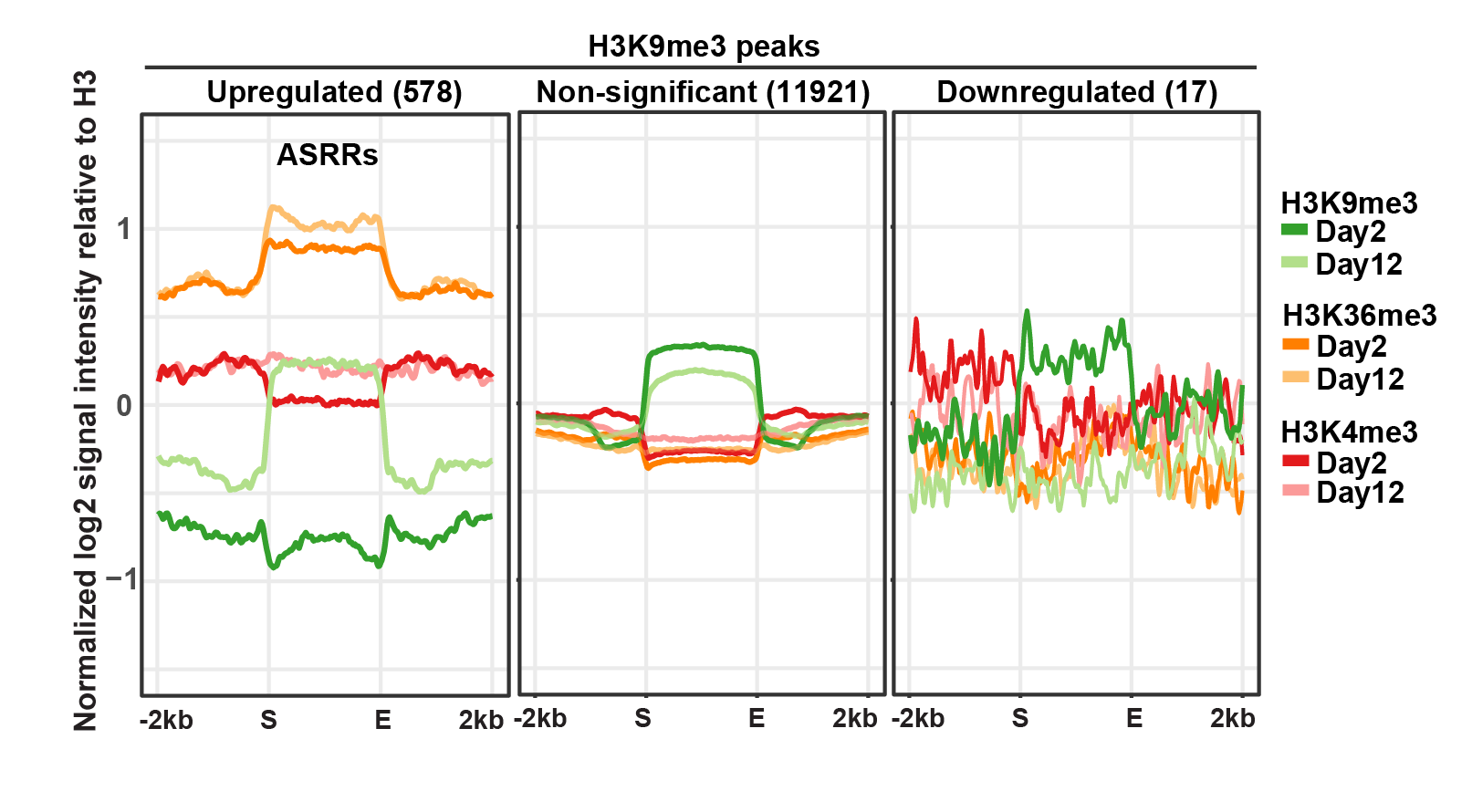

Supplement: S7 Fig — (A) Metaplots showing the average z-scores of H3K9me3, H3K4me3 and H3K36me3 ChIP-seq signal intensity normalized to H3 (log2) across H3K9me3 peak regions (± 2kb flanking regions: significantly upregulated (n = 578), significantly downregulated (n = 17) and non-significant (n = 11,921). H3K4me3 marks showed blurred boundaries and spreading into H3K9me3 peak regions at old age. H3K36me3 were depleted in non-significant H3K9me3 peaks but enriched in upregulated H3K9me3 peaks (ASRRs). (TIF) [file pgen.1009432.s007.tif]

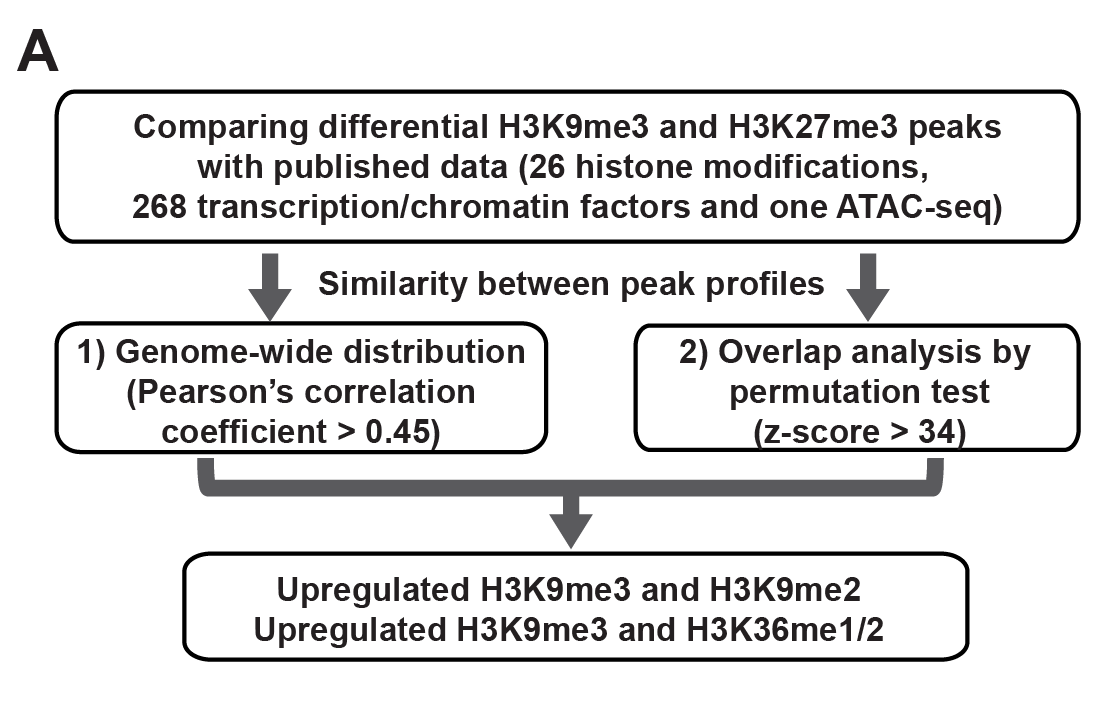

Supplement: S8 Fig — Differential H3K9me3 and H3K27me3 peaks were first compared with the publicly available datasets for their similarity in genome-wide distribution. Peak coverage in 50kb sliding windows was used to calculate pair-wise Pearson’s correlation coefficient. Next, we used the permutation test to compute the expected overlapping frequency between peak profiles. A z-score was given as a measure of the strength of association between peak profiles that deviate from expectation. Based on these analyses, upregulated H3K9me3 peak regions were strongly associated with H3K9me2 and H3K36me1/2 peak regions. (TIF) [file pgen.1009432.s008.tif]

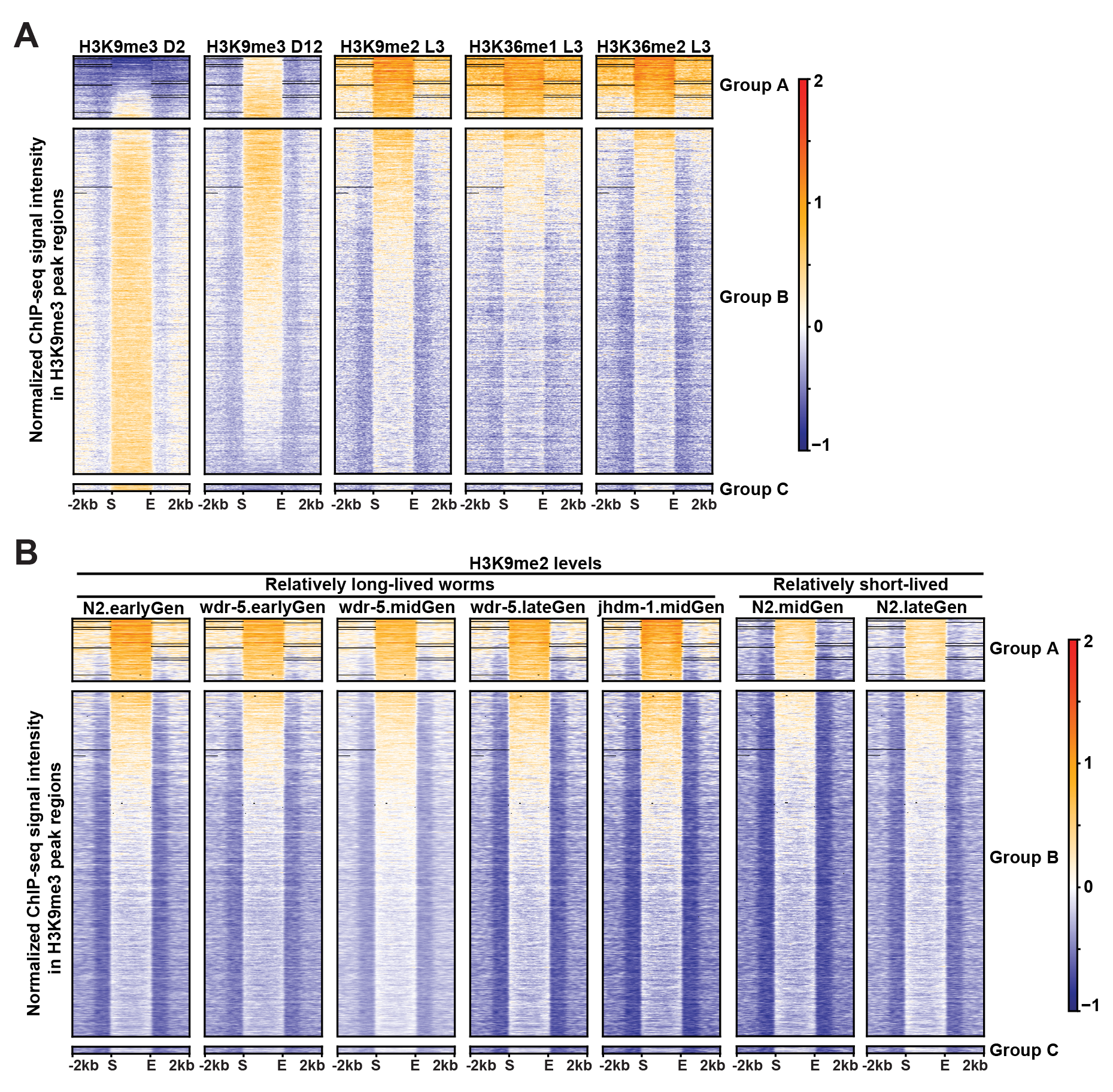

Supplement: S9 Fig — (A-B) H3K9me3 peaks were ranked by log2 fold change with age in descending order from top to bottom and separated into three groups based on log2 fold changes with age (A, > 1.25; B, 0.75~1.25; C <0.75). (A) Heatmap showing normalized ChIP-seq signal intensity (z-score) of H3K9me3 (young and old glp-1 mutants), H3K9me2 (N2 L3 larvae), and H3K36me1/2 (N2 L3 larvae) in H3K9me3 peak regions. (B) Normalized H3K9me2 ChIP-seq signal intensity (z-score) in N2 or wdr-5(ok1417) worms were generated by Lee et al. (10.7554/eLife.48498) and plotted here in heatmap format. ChIP-seq data were collected after thawing and growing worms for < 6 generations (earlyGen), 8–14 generations (midGen) or 20 generations (lateGen). (TIF) [file pgen.1009432.s009.tif]

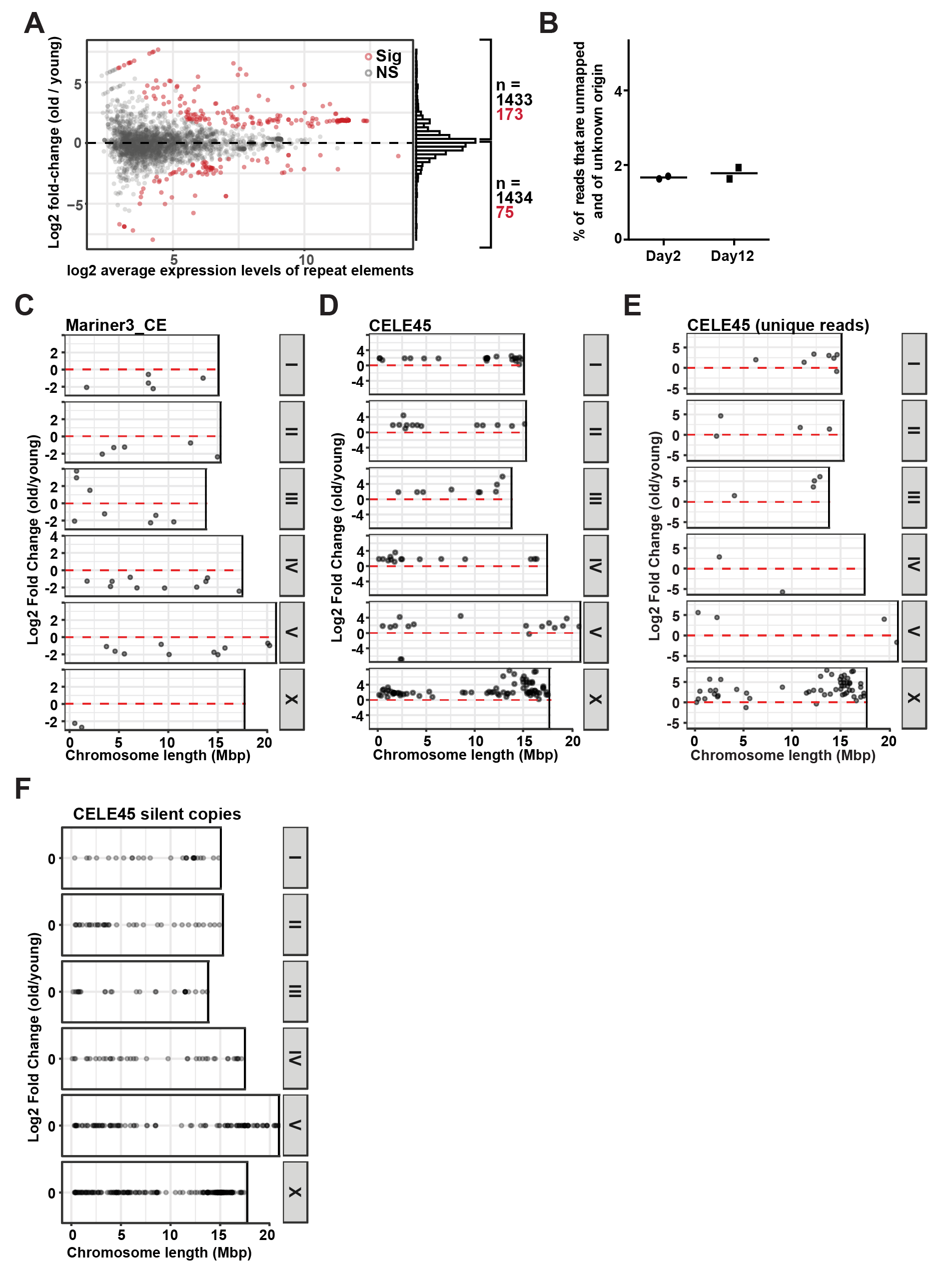

Supplement: S10 Fig — (A) MA-plot showing log2 fold changes of RNA expression of annotated repeats (dfam2.0) with age (old/young) in glp-1(e2141) worms. Repeat elements with significant expression changes are highlighted in red (Sig), and the rest are colored in grey (NS, not significant). (B) The percentage of unmapped reads with unidentified origin did not increase with age. (C) The genomic locations and RNA log2 fold changes with age of individual Mariner3_CE copies with detectable expression. (D and E) The genomic locations and log2 fold changes of RNA expression with age of individual CELE45 copies with detectable expression when multi-mapping RNA reads were included (D) or excluded (E). (F) The genomic locations of silent CELE45 copies. (TIF) [file pgen.1009432.s010.tif]

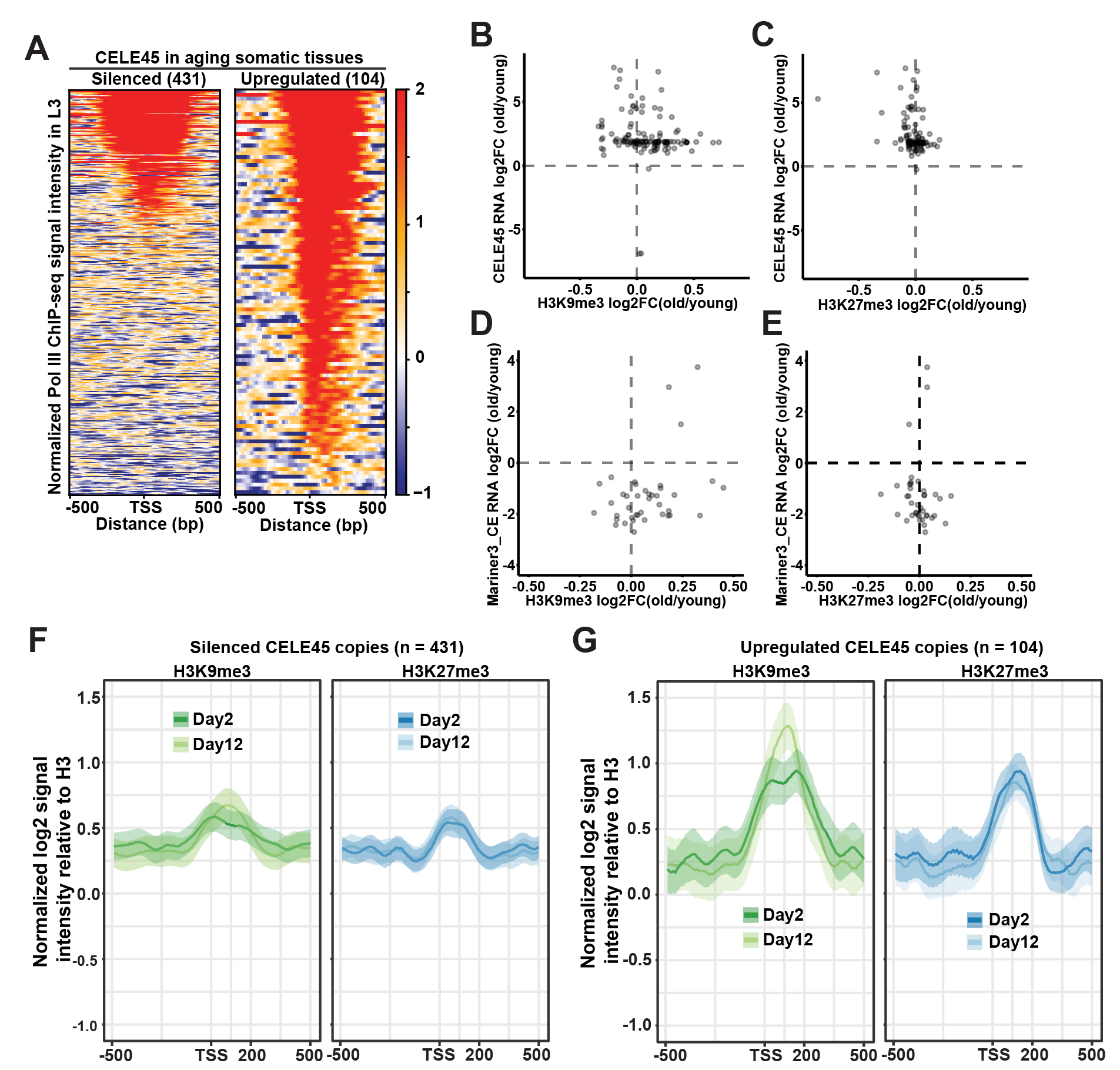

Supplement: S11 Fig — (A) Heatmap showing the Pol III ChIP-seq signal intensity (z-score) centered on the transcription start site (TSS) of silenced and expressed CELE45 copies. (B-E) Scatter plots showing the log2 fold changes of RNA expression and ChIP-seq signal intensity with age. Changes in repressive marks did not account for the overexpression of CELE45 copies and the downregulation of Mariner3_CE copies. (F-G) Metaplots showing the average z-scores of H3K9me3 and H3K27me3 ChIP-seq signal intensity normalized to H3 (log2) in silenced (F) and expressed (G) CELE45 copies. Expressed CELE45 copies were marked by higher levels of H3K9me3 and H3K27me3 in both young and old worms. (TIF) [file pgen.1009432.s011.tif]
