# Supplementary material for: Region-specific H3K9me3 gain in aged somatic tissues in Caenorhabditis elegans
Source: PLoS Genet. 2021 Sep 10;17(9):e1009432. doi: 10.1371/journal.pgen.1009432 (PMC8457455; doi:10.1371/journal.pgen.1009432)
Supplement: Attachment — Submitted filename: Rebuttal.pdf [file pgen.1009432.s022.pdf]

Reviewer#1
The following are included as constructive comments for the authors.

1. The authors introduced ASRRs in the discussion, but I would have found it helpful to have the term introduced earlier in the results, to make it clear which class of peaks are being referred to. For example, it takes some effort to realize that the 578 ASRRs are the same as the differentially-enriched peaks identified in line 234, and to remember that these represent less than 5% of all H3K9me3 peaks called.
    a. Furthermore, it is not clear whether ASRRs are solely peaks that exist in both young and aged populations (as is mildly indicated by language in lines 233-234), or whether they also include new peaks that are *only* found in aged populations (as would seem to be indicated by lines 441-442). This could be resolved by providing more detail in the results section.

We appreciate the reviewer's suggestion. In the revised manuscript, we introduced ASRRs (578 upregulated H3K9me peak regions) in the result section (line 286-287) and discussed it throughout the text. To improve clarity, we have also added the label "ASRRs" in the relevant figures (Fig 2B, 3A, 3C, S6A, S7A) and sentences in the main text.

2. If I'm interpreting the figures correctly, ASRRs are never examined as a class (but, as mentioned above, it'd be easier to know that for certain if ASRRs were named earlier and the term used throughout the results section). It seems like a missed opportunity to examine the genomic & transcriptomic landscape of ASRRs more closely. For example, how does gene expression change at these 578 peaks compared to all H3K9me3 peaks? Is it more or less drastic than shown in Fig S5A?

Do ASRRs in aged populations experience even higher levels of blurring & spreading of active modifications?

    a. Is the opposite true for the handful of down-regulated H3K9me3 peaks that make up the rest of the 595 significantly changed peaks?

We agree with the reviewer's suggestion and made the suggested revisions. We have made new plots (Fig 2A-B and S7) in which H3K9me3 peak regions were divided into three groups based on age-dependent changes in repressive marks (non-significant, significantly upregulated or significantly downregulated with age). We compared the levels of active histone marks (S7 Fig) and the RNA expression of genes (S2A-B) in the three groups of H3K9me3 peaks and non-peak regions.

3. It was hard to understand how Fig 2A-D is related to Fig 2E-F, in part because it wasn't clear how the genes shown in A-D overlap with the peaks used to examine expression in E & F. Are E and F only showing expression of peaks shown in the red lines? Providing N values might help clarify.

We agree with the reviewer's suggestion and revised the plots/text to improve clarity. Fig 2A-D include all genes whereas Fig 2E-F include only genes that were differentially expressed at old age. We have indicated the N number in all relevant figures as well.

4. Similarly, I would find it helpful for the metaplots to include the numbers of peaks used to generate them – it'd be easiest to see the N in the figure itself, but N values should at least be included in the figure legends. Including N values would help clarify whether we are looking at all peaks called, or drawing comparisons between a subset of specific peaks (like ASRRs).

We have added the N number in figures and/or figure legends.

5. The references in lines 489-490 don't seem to match with the text.

We have corrected the errors due to formatting issues.

6. Typos: Line 444 "K3K27me3," Line 447 "glp-1" should be italicized, and in the graph legend for Fig 2A "Upnregulated"

We have made revisions.

7. The writing is mostly clear, but there were numerous grammatical errors which would be easily caught by a proofreader. For example, "C. elegans" should be italicized throughout.

We have corrected the errors.

Reviewer #2

Major questions:

All these ChIP-seq experiments were done using glp-1 to focus on somatic changes. glp-1 mutants are long-lived, in addition to lacking a germline. Could it be that the increased longevity of the glp-1 mutants impacts the age-associated changes in chromatin that the authors observe? Is there any evidence that the age-associated changes in glp-1 mutants are recapitulated in other mutants that lack a germline but are not long-lived (glp-4, fer-1, etc)?

We agree with the reviewer's comment. *glp*-1 mutants were used to remove the germline and facilitate the harvest of somatic tissues. The age-associated changes observed in the *glp-1* mutants could be affected by the altered longevity associated with the *glp-1* mutants. As the reviewer suggested, we carried out preliminary investigation using *glp-4(bn2)* mutant, a temperature-sensitive germlineless mutant that has been reported to not exhibit altered longevity (Syntichaki et al., Nature 2007). Details of these results can be found at the end of this document on pg 9-10.

Line 159-164 the authors claim that the distribution of repressive marks in glp-1 is similar to wild-type and that the profiles for H3K9me3 and H3K27me3 between glp-1 adults and wild-type L3. They should show the data supporting these arguments, as they do for active marks in Fig S3B.

We appreciated the reviewer's suggestion. We have added two plots showing the profiles of H3K9me3 and H3K27me3 in wild-type L3 larvae in S3A Fig.

The authors claim that MDS analysis suggests that there are "substantial" age-dependent differences in H3K9me3, but "relatively minor" age-dependent variations in H3K27me3 samples (Fig 1A,B). It is not clear to me that this is an accurate description of the data, as MDS shows a simplified projection of distances between data in only two dimensions. How did the authors

evaluate the statistical significance of these differences to make this inference? In the MDS, does 2 dimensions capture the variance in the samples, and how did the authors evaluate this?

We agree with the reviewer's comment. The main purpose of the MDS plot is to visualize the clustering of samples rather than to test for the significance of the changes. We have revised the relevant sections and removed those sentences. Age-dependent changes in repressive marks were examined by differential analysis (line 218-226). Further analysis of the average changes in the enrichment of repressive marks were shown in the metaplots in Fig 1, Fig 3, S5 Fig and S7 Fig.

The data in Fig S3 seem to show that age-associated changes mostly change the magnitude of enrichment of both H3 modifications rather than a wholesale redistribution. This aspect of the data is lost when only the difference between young and old (as in Fig 1E) is shown. This could be misleading to a reader, so I think the authors should include Fig S3A in the main text. The authors should also include labels on the Y-axis of FIg 1E (and all similar plots) to indicate the magnitude of enrichment that is being shown. It would also be helpful to know which changes are statistically significant when data like these are shown. Finally, the authors should also be more circumspect in claiming "redistribution" in the text of the manuscript.

We agree with the reviewer's comment. We have used "region-specific gain and loss" to replace "redistribution" in the text.

The authors state that "most of the peak regions that lost repressive H3K9me3 or H3K27me3 marks were not statistically significant at FDR cutoff of 0.05" (line 235-236). But then they claim a correlation of increased gene expression at loci with "downregulated" repressive marks (Fig 2). I don't understand how the authors justify looking for correlations between changes in expression with non-significant changes in repressive marks.

We appreciate the reviewer's comment and have made revisions to improve clarity. In Fig 2E-F, we examined the RNA expression changes of differentially expressed genes that are associated with the repressive peak regions that showed statistically significant changes in H3K9me3 or H3K27me3 (line 250-255).

In Fig 2, the authors claim a statistically significant difference between curves based on the KS test. However, some of the differences are quite small. There are also large differences in the number of genes included in the two groups (especially in C and D). The authors need to explain how they ensured that the statistical test was sufficiently powered to detect the changes they report. If they perform these tests on independent data sets are the conclusions robust? Does the reciprocal comparison support these conclusions (ie looking for enrichment of H3K27me3 peaks in genes that are up- or down-regulated by RNAseq)? With the data provided it is nearly impossible for a reader to appreciate the robustness or biological significance of this conclusion.

We appreciate the review's comment. We have revised the plots in Fig 2A-D. Power analysis helps to determine whether the lack of statistical significance is due to the lack of

statistical power. It is not a concern when there is a statistically significant difference. The pair-wise KS tests between upregulated peaks, non-significant peaks, and non-peaks are all highly significant (p-value < 0.0001) despite some of the differences appear small. Lack of power did affect the downregulated repressive peak regions, which have small sample sizes and non-significant KS tests. The results are described in line 231-243 and the figure legends (Fig 2 and S4 Fig).

The authors state that "the majority of genes associated with repressive H3K9me3 and H3K27me3 peak regions were silent" but that "significantly upregulated repressive peak regions were preferentially associated with actively expressed genes" (line 276). This sentence is a bit confusing and should probably be edited for clarity;

We appreciate the reviewer's comment. We have made revisions to make it clear that the upregulated repressive peak regions only represent a small fraction of the total repressive peak regions. In this small faction of repressive peak regions, genes tend to be actively expressed.

moreover, I can't figure out where they show this with their data? Doesn't this statement contradict the data shown in Fig 2? Also in this paragraph, the authors claim that the fact the 'upregulated' peaks were lacking repressive marks in young animals supports the idea that these genes were expressed in younger animals. This seems like a circular argument. If you start by looking only at "new" peaks then it seems evident that the repressive marks had to be missing in the young animals, by definition. Does the RNAseq data support the assertion these genes are expressed at day 2 and not day 12?

We appreciate the reviewer's comment. We have found that most genes in upregulated repressive peak regions are transcriptionally active at both young and old time points by using our published RNA-seq data results (Line 273-277). We have revised the text to clarify this: The genes that were associated with upregulated repressive peaks and were transcriptionally active at young age remained transcriptionally active at old age.

The authors assert that "most of the actively expressed CELE45 copies in glp-1 adults had been actively transcribed starting at larval stages" and later conclude that the upregulation of this SINE is not associate with loss of repressive marks. It's not clear to me that this has anything to do with the age-associated changes in histone methylation that the authors focused on.

Previous studies in other organisms have found upregulation of transposons at old age correlates with the loss of repressive H3 marks. In this study, we found that despite high levels of repressive H3 markings in the CELE45 repeat regions (S11F and S11G Fig), many CELE45 copies remained transcriptionally active. These active CELE45 copies showed a significant increase in RNA expression at old age, which is not due to the loss of repressive H3 marks. This point is clarified in line 505-518.

Minor points

- Line 25 and 27 - consider changing "significant" to something else in order to avoid perception you are making a statistical argument.
We have replaced the "significant" in Line 26 with "evident". The "significant" in Line 29 does mean "statistically significant".

- Several different sentences in abstract start with "interestingly". The authors should edit the text to avoid overuse of conjunctive adverbs.
We have reduced the use of "interestingly" in the text.

- The authors should consider deleting the first paragraph of the Introduction. The second sentence of the introduction is vague and starts with a reference ("these epigenetic marks") that is confusing. Which marks? The first sentence is about deterioration of chromatin structures and epigenetic information, so the reference to epigenetic marks doesn't really make sense. The third sentence is redundant with the first sentence, and the last sentence doesn't really add any information.
We agree with the reviewer's comment and removed the first paragraph of the introduction.

- The text needs to be edited for grammar and clarity. There are several sentences that are awkward throughout the text. For example, the second sentence of the abstract (line 20) starts with "Dysregulated epigenome has been linked..." seems to be missing an article. I also noticed an over-abundance of conjunctive adverbs and a few places with random changes in tense.
We appreciate the reviewer's comment and have made revisions.

- It may be more correct to say that the dysregulation of repressive heterochromatin is associated (rather than "linked") with aging (line 69).
We appreciate the reviewer's comment and have made revisions (Line 62).

- Line 89, sentence beginning with "H3K27me3 is involved in..." needs a citation.
We have added citations (Line 86).

- Line 150, what is the "nevertheless" doing in this sentence?

We have removed the "nevertheless".

- What do the numbers on the top left of each trace in Fig S3 mean?

We have revised the figure legend to clarify that those numbers indicate the y-axis range (z-scores).

- The authors say that H3K27me3 is enriched in H3K9me3-depleted central regions of chr II and

IV, but data in Fig S3 seems to show that there is also more H3K27me3 signal in the middle of chr V. It's not clear why this was not mentioned by the authors.

We agree with the reviewer's comment. We have removed the "II and IV" because H3K27me3 is more enriched in the middle of all chromosomes than H3K9me3.

- The authors merged neighboring peaks if they were within 5 kb (line 152-153). I don't understand the justification for doing this - are there data showing that peaks are generally this large in C. elegans? What is the average size of a peak in the author's data without this manipulation?

The main reason is to adjoin neighboring peaks which in fact represent one long enrichment domain (see S2A Fig, Line 143-148). The threshold (5kb) were empirically determined (S2B/C Fig).

- The authors say that the majority of peaks marked with H3K9me3 and H3K27me3 are not expressed (paragraph starting at line 216). It is not clear to me how they did this analysis.

(Line 165-170) We first identified the set of genes that overlapped with the repressive peak regions. For these genes, we examined their RNA expression levels by using our previously published RNA-seq data (Pu et al. 2018). We then calculated the fraction of genes that have zero RNA read counts.

- The authors claim that the observation that there is not much overlap in where age-associated changes in H3K27me3 and H3K9me3 occur suggests that "different mechanisms" contribute to the age-dependent changes. I'm not sure I follow this logic. I would expect that modifications at K27 and K9 would use distinct writers and erasers, regardless of effects of aging, so I'm not sure what "mechanisms" they are referring to here.

We appreciate the reviewer's comment and have revised the relevant sentences for clarity. In *C. elegans*, there is a strong association between H3K9me3 and H3K27me3 in repressive heterochromatin (Ho et al. Nature 2014). Worm-specific co-incidence of H3K9me3 and H3K27me3 is observed in repressive peak regions established at young age but not in repressive peak regions newly formed at old age. There might be specific biological processes regulated the co-localization of repressive H3K9me3 and H3K27me3 which become dysregulated at old age.

- Line 248 there is a reference written as "(2)" in parenthesis instead of brackets; also I don't think the previous findings referred to here should reference to Sen et al.

We have corrected the wrong reference.

- Line 248 it seems there is an extra "Fig" in the parentheses.

We have revised the text.

- In Fig2A the label for "upregulated" is misspelled.

We have corrected the error.

- The authors use "upregulated" and "downregulated" to refer both to gene expression and accumulation or loss of repressive marks, and this gets confusing at points. The authors need to choose another way to refer to changes in ChIP-seq peaks to avoid ambiguity.

increased and decreased expression

We appreciate the reviewer's comment and have made revisions. We have used "upregulate and downregulate" to describe the changes in repressive marks and "increase/decrease" to describe the changes in RNA expression.

- Line 277: the authors refer to genes that are "silent". Do they mean that in RNAseq these transcripts were not detected?

Genes with no detectable RNA reads (zero read count) were referred to as "silent".

- it would be useful to know the experimental conditions for the publically available ChIP-seq and ATAC-seq data sets the authors used to compare to the differential repressive peaks (section starting at bottom of pg 12). A table showing life stage, genotype, reference, and other relevant info would be very helpful here.

We have added a new table (S6 Table).

- Line 537: is "protease K" supposed to read "proteinase K"?

We have corrected the mistake.

Reviewer #3

Major Comment:

The only major comment I have is that, as the experimental setup utilizes day 2 and day12 glp-1 (e2141) animals grown at 25C and lacking a germline, it would be important to know if changes that occur during normal aging at 20C recapitulate what is observed in these germline-less mutants. This can be done by assaying by ChIP-PCR H3K9/K27 me3 occupancy at a few randomly selected loci (e.g., those in Fig S6G) which display significant changes in glp-1 animals. These data would be informative as they are more true to what happens during normal aging, even though they may be complicated by the presence of germline cells in these wild-type animals.

We appreciate the reviewer's comment. In N2 animals, approximately two-thirds of the cells are germline cells and we may not be able to detect the epigenetic changes in somatic

cells. We agree that it would be informative to investigate the epigenetic changes during normal aging in wild-type animals. One feasible approach is to isolate somatic cells from wild-type animals by FACS followed by epigenetic profiling. These will be our long-term goals and need further technical development, which is beyond the scope of this study.

Minor comments:
1. Lines 24: typo (repeated word).

We have corrected the typo.

Summary

Here, we compared the H3K9me3 profile produced by CUT&RUN-seq (2 replicates) in the germlineless *glp-4(bn2)* mutant with the H3K9me3 profile produced by ChIP-seq in the germlineless *glp-1(e2141)* mutant (Fig 1A). At old age, the average H3K9me3 enrichment across all H3K9me3 peak regions were reduced in both strains (Fig 1B). The loss of H3K9me3 enrichment in peak regions was more prominent in *glp-4* than in *glp-1* mutants (Fig 1A and 1B).

H3K9me3 peak regions were divided into three groups based on the fold change (old/young) of H3K9me3 enrichment in the *glp-1* mutants (>1.25 in group A, 0.75~1.25 in group B and <0.75 in group C). The data suggested an age-dependent increase in H3K9me3 enrichment in the Group A peaks in the *glp-4* mutant, similar to that in the *glp-1* mutant (Fig 1C). To more clearly demonstrate this, we examined the ASRRs, which are regions that showed significant increases in H3K9me3 at old age in the *glp-1* mutants, the data suggested similar age-dependent increase in the H3K9me3 levels in the *glp-4* mutants (Fig 1D).

Taken together, *glp-4* mutants showed a global loss of H3K9me3 enrichment in peak regions, similar to that of *glp-1* mutants. Peak regions (Group A in Fig 1C and ASRRs in Fig 1D) that gained H3K9me3 in the *glp-1* mutants also showed increased H3K9me3 enrichment in the *glp-4* mutants. We therefore concluded that our findings in the *glp-1(e2141)* mutant, which are extensively discussed in the manuscript, are likely representative of germlineless strains and not due to the specific mutation in the *glp-1* gene. However, due to time constraints and that the primary author Cheng-Lin Li has already begun a new position as a Bioinformatician at SOPHiA Genetics since March, we are not able to conduct additional replicates and therefore do not plan to include the preliminary CUT&RUN data in the manuscript.

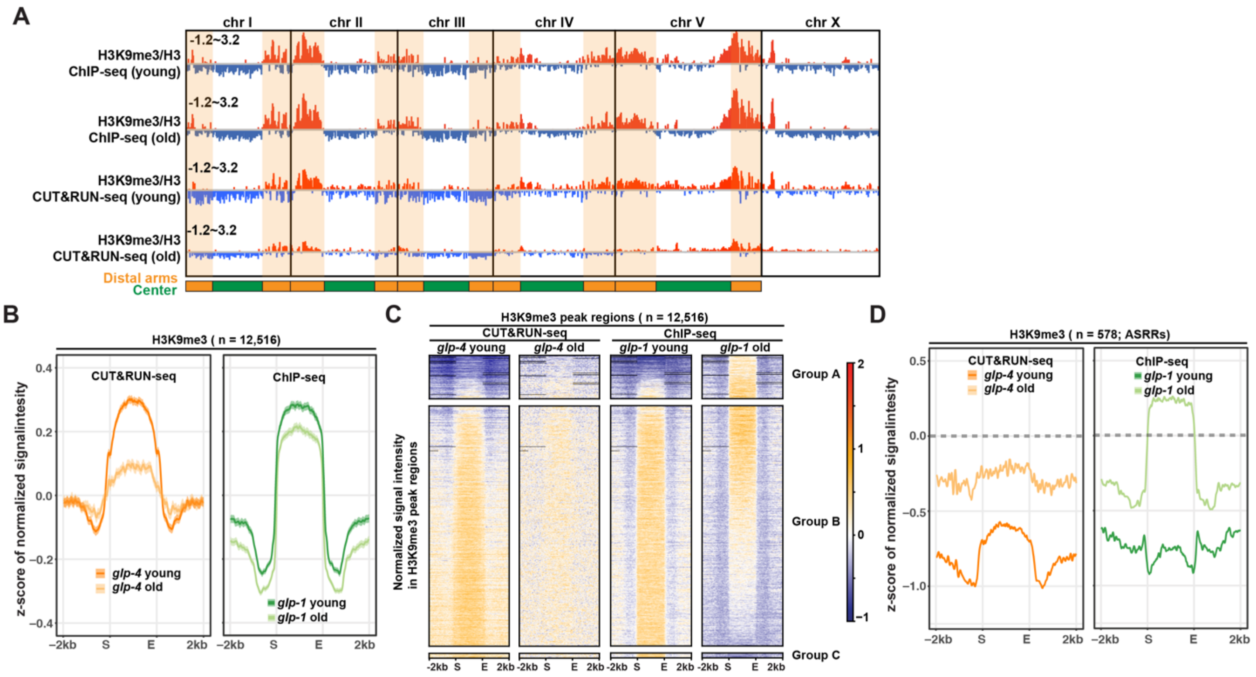

Fig 1. H3K9me3 profiles in *glp-4(bn2)* and *glp-1(e2141)* mutants. (A) IGV browser screenshot showing z-scores of H3K9me3 signal intensity normalized to H3 in young and old worms. H3K9me3 profiles in *glp-1* and *glp-4* mutants were generated using ChIP-seq or CUT&RUN-seq respectively. **(B)** Metaplots showing the average z-scores of H3K9me3 signal intensity normalized to H3 (log_2) across H3K9me3 peak regions (± 2kb flanking regions) in *glp-4* or *glp-1* mutants. **(C)** H3K9me3 peaks in *glp-1* mutants were ranked by log_2 fold change with age in descending order from top to bottom and separated into three groups based on log_2 fold changes with age (A, > 1.25; B, 0.75~1.25; C <0.75). CUT&RUN signals in the corresponding regions in *glp-4* mutants showed similar patterns. **(D)** Metaplots (green) showing the average z-scores of H3K9me3 signal intensity normalized to H3 (log_2) in peak regions (± 2kb flanking regions) that significantly upregulated at old age in *glp-1* mutants (i.e. the ASRRs). Metaplots (orange) for the *glp-4* mutants were generated similarly using the H3K9me3 CUT&RUN-seq data in the corresponding regions.

Methods

For the CUT&RUN-seq experiment, the *glp-4* mutants were grown and harvested exactly as those for the *glp-1* mutants. Briefly, *glp-4(bn2)* worms were maintained at 16°C. To prepare age-synchronous populations of germlineless *glp-4(bn2)* adults, eggs laid by day 1 adults over a period of 4-8 hours were collected and grown at 25°C on 15-cm nematode growth medium (NGM) plates. We harvested two biological replicates of day 2 (young) and day 12 (old) adults to profile H3 and H3K9me3 enrichment by CUT&RUN-seq (Skene *et al.*, eLife 2017). First, we washed worms off plates with M9 buffer and dissociated the whole worms into cell suspensions followed the protocol developed by Dr. Murphy's group (Kaletsky *et al.*, Nature 2016). The cell suspensions were incubated with the same anti-H3 and anti-H3K9me3 antibodies used for ChIP-seq experiments in the manuscript. Next, cell suspensions were incubated with the protein A-Micrococcal Nuclease (pA/MNase) fusion protein. DNA associated with the antibody-pA/MNase complexes was then digested, released, and purified for library preparation.
