# Supplementary material for: Region-specific H3K9me3 gain in aged somatic tissues in Caenorhabditis elegans
Source: PLoS Genet. 2021 Sep 10;17(9):e1009432. doi: 10.1371/journal.pgen.1009432 (PMC8457455; doi:10.1371/journal.pgen.1009432)
Supplement: Attachment — Submitted filename: PGENETICS-D-21-00229 review.docx [file pgen.1009432.s021.docx]

**Review:** Locus-specific H3K9me3 gain in aged somatic tissues in *Caenorhabditis elegans*

In this manuscript, the authors used ChIP-seq to characterize age-related changes in two canonical markers of repressive heterochromatin, H3K27me3 and H3K9me3. They found that aging caused drastic changes at the boundaries between H3K9me3 and active modifications. Similarly, comparing called peaks in their ChIP-seq with publicly available data (including dozens of other histone modifications), they found that H3K9me3 peaks correlated strongly with peaks called in H3K9me2 and H3K36me1/2. They then correlated peaks of repressive modifications with RNA-seq from an earlier study to identify transcriptional changes. Given the role of heterochromatin (H3K9me3 in particular) in silencing repetitive elements, it was surprising that they observed no global de-repression of repeats. However, upon examining the few repetitive elements that were upregulated with age, they identified a retrotransposon family that was overrepresented among differentially expressed repeats, CELE45, although it did not appear to be de-repressed due to locus-specific changes in H3K27me3 or H3K9me3.

This study provides an important resource for the field. Previous studies, in *C. elegans* and other organisms, have characterized global changes in these modifications with age. However, as with all histone modifications, bulk levels of any particular modification may serve as a poor indicator of functional consequences. Examining genomic changes over time in a locus-specific manner provides a more meaningful understanding of potential consequences, and has the potential to identify unexpected relationships that may affect aging. The identification of CELE45 as an aging-specific transcriptomic change highlights an interesting future direction, especially its association with a stress-response.

However, the manuscript would benefit greatly from a few more analyses and a more scholarly presentation of the introduction & discussion. The two halves of the paper (ChIP-seq and transcriptomics/CELE45) feel disjointed, particularly because the central hypothesis set up in the introduction (that changes in histone modifications affect aging by altering expression) was not strongly supported. Interesting observations are made, but not explored. For example, the relationship between H3K9me3 and other active modifications indicates some interesting biology, but the relationship between blurred domain boundaries and gene expression is not examined: rather than focusing only on genes with overlapping H3K9me3 peaks, they could examine changes in gene expression by examining changes at boundary domains.

Furthermore, recent work in the field has identified important distinctions between H3K9me2 and H3K9me3 in *C. elegans* genomes. The Ahringer lab has shown that canonical heterochromatin proteins like HPL-2 correlate strongly with H3K9me2 peaks, but *not* H3K9me3 peaks (McMurchy et al 2017). The distinction between H3K9me2 and H3K9me3 may account for the minor effect on transcription at significantly-changed peaks. However, equating H3K9me3 with heterochromatin seems disingenuous, and may obscure some interesting chromosomal biology. Given that the authors present a model in which H3K9me2 is converted to H3K9me3 in aged populations, they should address how this loss of heterochromatic H3K9me2 (and blurring of domain boundaries) fits with what’s been observed elsewhere (or perhaps differs from prior observations).

Overall, the experiments conducted in this paper were well-founded, and the conclusions are generally supported by the data. Revision of the writing and a few additional genomic analyses will provide much-needed context. This manuscript will be of broad interest to researchers who study aging, and of high interest to those in the *C. elegans* chromatin community.

The following are included as constructive comments for the authors.

1. The authors introduced ASRRs in the discussion, but I would have found it helpful to have the term introduced earlier in the results, to make it clear which class of peaks are being referred to. For example, it takes some effort to realize that the 578 ASRRs are the same as the differentially-enriched peaks identified in line 234, and to remember that these represent less than 5% of all H3K9me3 peaks called.
   a. Furthermore, it is not clear whether ASRRs are solely peaks that exist in both young and aged populations (as is mildly indicated by language in lines 233-234), or whether they also include new peaks that are *only* found in aged populations (as would seem to be indicated by lines 441-442). This could be resolved by providing more detail in the results section.
2. If I’m interpreting the figures correctly, ASRRs are never examined as a class (but, as mentioned above, it’d be easier to know that for certain if ASRRs were named earlier and the term used throughout the results section). It seems like a missed opportunity to examine the genomic & transcriptomic landscape of ASRRs more closely. For example, how does gene expression change at these 578 peaks compared to all H3K9me3 peaks? Is it more or less drastic than shown in Fig S5A? Do ASRRs in aged populations experience even higher levels of blurring & spreading of active modifications?
   a. Is the opposite true for the handful of down-regulated H3K9me3 peaks that make up the rest of the 595 significantly changed peaks?
3. It was hard to understand how Fig 2A-D is related to Fig 2E-F, in part because it wasn’t clear how the genes shown in A-D overlap with the peaks used to examine expression in E & F. Are E and F only showing expression of peaks shown in the red lines? Providing N values might help clarify.
4. Similarly, I would find it helpful for the metaplots to include the numbers of peaks used to generate them – it’d be easiest to see the N in the figure itself, but N values should at least be included in the figure legends. Including N values would help clarify whether we are looking at all peaks called, or drawing comparisons between a subset of specific peaks (like ASRRs).
5. The references in lines 489-490 don’t seem to match with the text.
6. Typos: Line 444 “K3K27me3,” Line 447 “glp-1” should be italicized, and in the graph legend for Fig 2A “Upnregulated”
7. The writing is mostly clear, but there were numerous grammatical errors which would be easily caught by a proofreader. For example, “C. elegans” should be italicized throughout.
